# Body Mass Index and Nutritional Dynamics as Predictors of Survival in Patients with Oropharyngeal Squamous Cell Carcinoma Undergoing Chemoradiation

**DOI:** 10.3390/jcm15208142

**Published:** 2026-10-20

**Authors:** Julian Emanuel Bonea, Monica Emilia Chirila, Renata Zahu, Nicolae Todor, Ștef Ionuț Andrei, Daniela Elena Sturzu, Andrei Țenț, Vlad Manolescu, Gabriel Kacsó

**Affiliations:** 1 Department of Human Anatomy and Embriology, Iuliu Hatieganu University of Medicine and Pharmacy, 400015 Cluj-Napoca, Romania; julian.eman.bonea@umfcluj.ro; 2 Department of Oncology, Iuliu Hatieganu University of Medicine and Pharmacy, 400015 Cluj-Napoca, Romania; chirila.monica.emilia@umfcluj.ro (M.E.C.); renata.zahu@umfcluj.ro (R.Z.); 3 Department of Radiation Oncology, Amethyst Radiotherapy Center Cluj, 407280 Floresti, Romania; 4 Department of Statistics, Ion Chiricuta Institute of Oncology, 400015 Cluj-Napoca, Romania; todor@iocn.ro; 5 General Medicine Faculty, Iuliu Hatieganu University of Medicine and Pharmacy, 400015 Cluj-Napoca, Romania; stefan.ionut.andrei@umfcluj.ro; 6 Department of Oral and Maxillo-Facial Surgery, Faculty of Medicine and Pharmacy, University of Oradea, 410073 Oradea, Romania; tent_andrei@yahoo.com; 7 Department of Medical Oncology, Amethyst Radiotherapy Center Cluj, 407280 Floresti, Romania; 8 Department of Medical Oncology, Emergency County Hospital, 400347 Cluj-Napoca, Romania

**Keywords:** oropharyngeal cancer, body mass index, chemoradiation, weight loss, prognosis

## Abstract

**Background/Objective:** Body mass index (BMI) and treatment-related weight variation have been increasingly investigated as potential prognostic factors in head and neck oncology. However, their role in patients with oropharyngeal squamous cell carcinoma (OPSCC) undergoing concomitant chemoradiation (CRT) remains incompletely defined, particularly in real-world Eastern European populations. The aim of this study was to evaluate the prognostic significance of pretreatment BMI and treatment-induced weight variation in patients with OPSCC treated with curative-intent CRT and to assess their association with survival outcomes. **Methods:** This single-centre retrospective cohort study evaluated consecutive patients with OPSCC treated with CRT at a Romanian regional oncology centre between 2015 and 2023. Key exclusions were prior surgery or neoadjuvant chemotherapy, palliative care, and secondary malignancies. Clinical variables, baseline comorbidity burden (via Age-Adjusted Charlson Comorbidity Index), and body mass index (BMI) dynamics across treatment timepoints—including pre- and post-CRT BMI, ΔBMI during CRT, and weight loss thresholds ≥5% and ≥10%—were assessed. Overall survival (OS) was analysed using Kaplan–Meier curves, log-rank tests, and Cox proportional hazards multivariate regression to determine independent prognostic factors (*p* <0.05). **Results:** Among 114 patients analysed (median age 61; 80.7% male; median follow-up 52 months), 55.2% experienced ≥5% weight loss during chemoradiotherapy (CRT), and 22.8% lost ≥10%. On univariate analysis, significant predictors of reduced 5-year overall survival (OS) included age ≥65 years (HR 2.47, *p* = 0.01), CRT duration ≥ 47 days (63.2% vs. 92.3%; HR 2.52, *p* = 0.05), weight loss ≥ 10% (50.0% vs. 71.6%; HR 2.28, *p* = 0.03), pre-CRT BMI < 24 kg/m^2^ (HR 0.50, *p* = 0.04), post-CRT BMI < 24 kg/m^2^ (HR 0.40, *p* < 0.01), and a BMI reduction > 6% (HR 0.50, *p* = 0.03). On multivariate Cox regression, only age at diagnosis and CRT treatment duration were validated as independent prognostic factors for OS. **Conclusions:** Weight loss during concomitant CRT and low body mass index were associated with impaired 5-year OS in patients with oropharyngeal squamous cell carcinoma. However, after multivariate adjustment, age at diagnosis, and treatment duration were the sole independent prognostic factors.

## 1. Introduction

Head and neck cancers (HNC) represent a significant global burden, with almost one million new cases reported each year [1]. Oropharyngeal cancers (OPCs) represent the fourth most frequent anatomical site of HNC worldwide, and the third in Romania, after oral cavity and larynx cancers [2,3]. According to the Globocan 2024 report for Romania, OPCs account for 1300 of the 6000 new HNC patients diagnosed each year [3].

Chemoradiation (CRT) is widely used for treating HNC in various clinical scenarios [4]. Despite technological improvement which improved dose conformality with a subsequent decrease in the frequency and severity of side effects, up to 75% of the patients still experience G2–3 dysphagia, and more than 50% develop oral or pharyngeal mucositis [5], which can lead to food intake reduction and significant weight loss [6].

The impact of weight loss on patient’s outcome may vary according to the pre-treatment starting point. A recent systematic review which included eight studies concluded that pretreatment weight loss of > 10% was predictive of worse OS and disease-specific survival (DSS). By contrast, the evidence for the impact of weight loss during or after treatment remains inconsistent [7].

A pre-treatment BMI > 25 kg/m^2^ has been identified as a favourable prognostic factor for overall survival in HNC patients in large retrospective cohorts [8]. A 2023 systematic review and meta-analysis showed that HNC patients with a BMI of 25–30 kg/m2 had a better overall survival (OS), but those with a BMI > 30 kg/m2 did not [9].

Patients diagnosed with OPC receiving radiotherapy have a higher prevalence of critical weight loss compared to other sites, according to the meta-analysis by Zhang et al. [10]. However, currently published data on the impact of BMI variations in this group of patients remans scarce and conflicting [10,11]. Moreover, real-world data from Eastern European countries are still lacking.

The aim of this study was to evaluate body weight changes in patients with OPC receiving CRT with curative intent in a Romanian cancer centre and to assess the prognostic value of BMI and weight loss on long-term clinical outcomes.

## 2. Material and Methods

### 2.1. Patient Selection

We included consecutive patients diagnosed with oropharyngeal squamous cell carcinoma (OPSCC) who received concomitant CRT in a regional radiotherapy centre between January 2015 and December 2023, based on the multidisciplinary tumour board recommendation. We excluded non-squamous histological subtypes, patients who received palliative RT or had other malignancies (previously treated, synchronous or metachronous), and those with prior surgical treatment or neoadjuvant chemotherapy. All patients were offered nutritional specialist counselling. The follow-up was systematically scheduled “in house”, regularly at 2, 6, 12, 18, 24, 36, 48, and 60 months.

The study protocol was approved by the University of Medicine and Pharmacy Cluj-Napoca Ethics Committee (no 234/22.11.2024) and complies with the 1964 Declaration of Helsinki and its latest revision adopted by the World Medical Association in October 2024. Patient data were retrospectively retrieved from the institutional electronic database and anonymized prior to analysis. All patients have signed an informed consent stating that their clinical data can be used for research purposes at their initial presentation in the clinic.

### 2.2. Clinical Variables and Anthropometric Assessment

Patients’ sex refers to sex assigned at birth, and age represents the age at diagnosis. Smoking status was simplified into two categories. Current smoker was defined as smoking when CRT started or abstinent for less than 6 months prior. Staging was carried out according to the AJCC Version 9 criteria.

Patient comorbidity burden at baseline was quantified using the Age-Adjusted Charlson Comorbidity Index (ACCI), calculated via the MDCalc platform [12]. The ACCI evaluates 17 comorbid conditions weighted from 1 to 6 points based on relative mortality risk, with additional point weighting applied for age (+1 point for every decade above 40 years). Comorbidity data were manually extracted through retrospective review of electronic health records. Total ACCI scores were categorized into two risk tiers (<5 for low to moderate comorbidity burden, and ≥5 for high comorbidity burden).

Body weight and height data were collected from medical records. Weight at beginning and end of CRT was measured by the clinic’s nutrition specialist or self-assessed by the patient. The patients were instructed to use the same measuring device—at home or in the clinic—to avoid bias.

Body mass index (BMI) was calculated as weight in kilograms divided by height in meters squared (kg/m^2^). According to World Health Organization classification criteria, patients were categorized as underweight (<18.5), normal weight (18.5–24.9), overweight (25.0–29.9), obesity class I (30.0–34.9), obesity class II (35.0–39.9), or obesity class III (morbid) (≥40.0) [13].

### 2.3. Study Design

This is a single-centre retrospective cohort study. Two body weight measurements were analysed for each patient: at CRT start (Day1) and at CRT end (last day). We used univariate analysis to test the predictive value for OS of clinical variables such as sex, age, clinical stage, comorbidity score, and smoking status, along with body weight variations. For each of the three above-mentioned timepoints, we evaluated three BMI categories: normal or underweight (<25), overweight (≥25 and <30), and obese (≥30). We calculated a ΔBMI, which represents the difference from the BMI value at the end and at the beginning of CRT. We also analysed two pairs of patients’ categories: those who had stable or increased weight during CRT, those who lost <5% or ≥5%, respectively, or those who lost <10% or ≥10% of their weight; thresholds were integrated in the WHO performance status assessment. We subsequently applied multivariate analysis to test if the variables keep their prognostic value.

The missing categorial data was labelled as unknown (or not-available, NA) and treated as a separate category.

### 2.4. Statistical Analysis

Patients were censored at the last control (in person or by telemedicine) if no medical information could be retrieved for more than 1 year. We evaluated time-to-event data using overall survival (OS), defined as the interval from the start of treatment to death or last contact. We estimated survival curves with the Kaplan–Meier method, comparing them via the log-rank test, and used Student’s *t*-tests to assess mean differences between groups. To identify the values that are generating the lowest *p*-values, we used the 2024 MedCalc version 23 software modulation (MedCalc software Ltd., Ostend, Belgium) with 1-unit increments as cut-offs for body weight variables if the remaining comparator group had more than 10 patients. After conducting univariate screening, we performed multivariate modelling using Cox proportional hazards analysis. We set the significance level at 0.05 with 95% confidence intervals. Statistical calculations were carried out in Excel 2021 and StatView version 11 [14,15].

## 3. Results

### 3.1. Patients

We retrospectively analysed data from 114 consecutive OPSCC patients with a median age of 61, ranging from 38 to 78. Their baseline clinical characteristics are summarized in Table 1, as well as their tumour stage and HPV status. The patients were predominantly male (N = 92; 80.7%), and the most prevalent anatomical site was the tonsil (N = 69; 60.5%). More than half of the patients were smoking at the time of CRT start (N = 64, 56.1%) and had a low/mild score of comorbidities (N = 81; 71.1%). Unfortunately, the HPV status of 57% of our patients was unknown (Table 1).

The main treatment scenarios are depicted in Table 2. High-dose Cisplatin (N = 65; 57.0%) or weekly Cisplatin (N = 26; 22.8%) were the preferred concurrent systemic therapy. The standard 70 Gy/35 fr scheduled was received by most of the patients (N = 97; 86.6%). The median CRT overall treatment time was 48.5 days, ranging from 42 to 70 days. Most of the time, the duration was from 47 to 50 days (N = 83; 72.8%). Three patients had longer duration due to acute health issues (COVID 19, renal failure, or electrolyte imbalance), which was compensated by an additional dose (Table 2).

The median follow-up was 52 months, ranging from 17 to 121 months. At the time of the analysis, 72 (63.2%) patients were alive, and 42 (36.8%) were deceased.

### 3.2. Weight Variation

**Figure 1:**
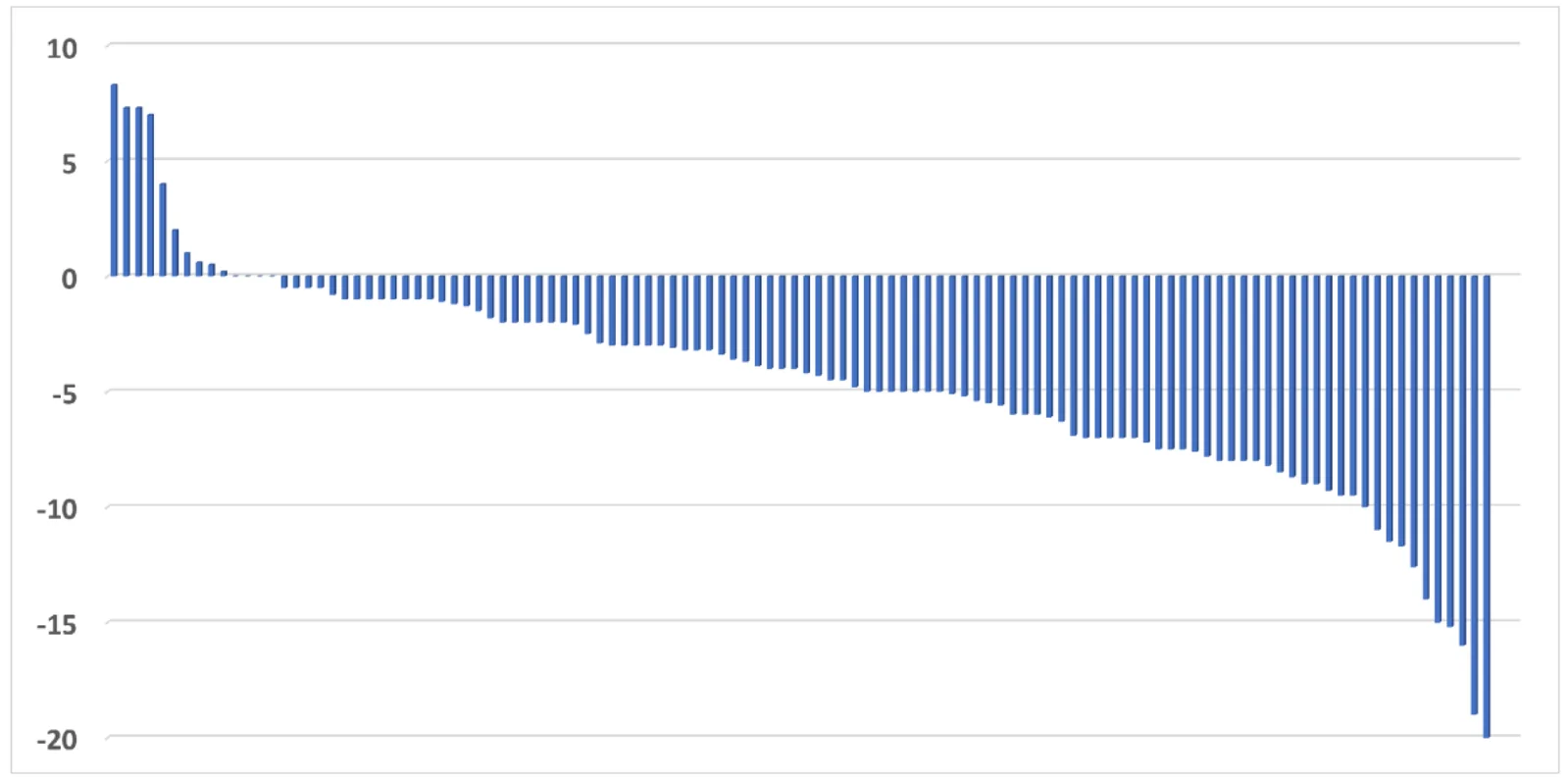
Histogram of individual weight variation between the start and the end of CRT (N = 114). The numerical values represent kg.

There was an overall decrease of BMI during CRT, as most of the patients lost weight during CRT (Figure 1). The median weight pre-CRT was 75 kg (38–120), and post-CRT it was 70 kg (36–114). Sixty-three patients (55.2%) lost ≥5%, and 26 patients (22.8%) lost ≥10% of their pre-CRT weight (Figure 1).

The median value of pre-CRT BMI was 25.4 (15.8–37), and post-CRT it was 23.9 (15.0–35.2). The median variation was −1.4 Kg/m^2^ (ranging from −6.5 to +4.3).

### 3.3. Clinical Factors That Influenced OS

We included several variables in the univariate analysis of predictive factors.

A better survival at 5 years was observed in women (69.3% vs. 65.5%), younger patients (71.8% vs. 57.2%), lower stage cases (88.2 for stage II, 66.2% for stage III, and 61.6% for stage IV), and HPV-positive cases (72.7% vs. 55.0%). However, statistical significance was observed only in those aged less than 65 years compared to those aged 65 or more (HR 2.47, 95%CI 1.96–3.11, *p* = 0.01).

Patients with less comorbidities and non-smokers had a better prognosis, but the values were close and non-significant. The 5-year OS was 66.6% for a Charlson Comorbidity index of less than 5, compared to 65.3% for a higher score. Those who were not smokers at the time of CRT had a probability of being alive at 5 years of 68.0% compared to 66.6% for smokers.

A treatment duration longer than 47 days was associated with a worse survival (63.2% vs. 92.3%, HR 2.52, 95% CI 1.62–3.90, *p* = 0.05) (Table 3).

### 3.4. Prognostic Value of Weight Variation

Patients with a higher percentage of weight loss had a worse 5-year OS. The threshold of 10% was associated with statistical significance (50.0% vs. 71.6%, HR 2.28, 95% CI 1.70–3.05, *p* = 0.03).

When analysing pre-CRT BMI categories, better survival was found in overweight patients (71.2%) compared to obese (66.7%) and to those with a BMI below 25 (61.9%). In the post-CRT setting, those with a BMI over 30 had the highest values of 5-year OS (83.3%), followed by those who were in the range of 25–29.9 (74.0%). Similar to the pre-CRT setting, the BMI under 25 was associated with the lowest OS (61.1%).

Patients who had a decrease of more than 1.5 points in BMI values after CRT compared to the pre-CRT BMI had a worse prognosis at 5 years (59.8% vs. 74.3%). However, the difference was not statistically significant (*p* = 0.06).

A BMI value of 24 was identified as a statistically significant threshold for both pre-CRT (HR 0.50, 95% CI 0.40–0.63, *p* = 0.04) and post-CRT settings (HR 0.40, 95% CI 0.33–0.48, *p* <0.01). Additionally, a decrease of more than 6% BMI during CRT was predictive of a lower OS (HR 0.5, 95% CI 0.41–0.61, *p* = 0.03) (Table 4).

### 3.5. Multivariate Analysis

**Figure 2:**
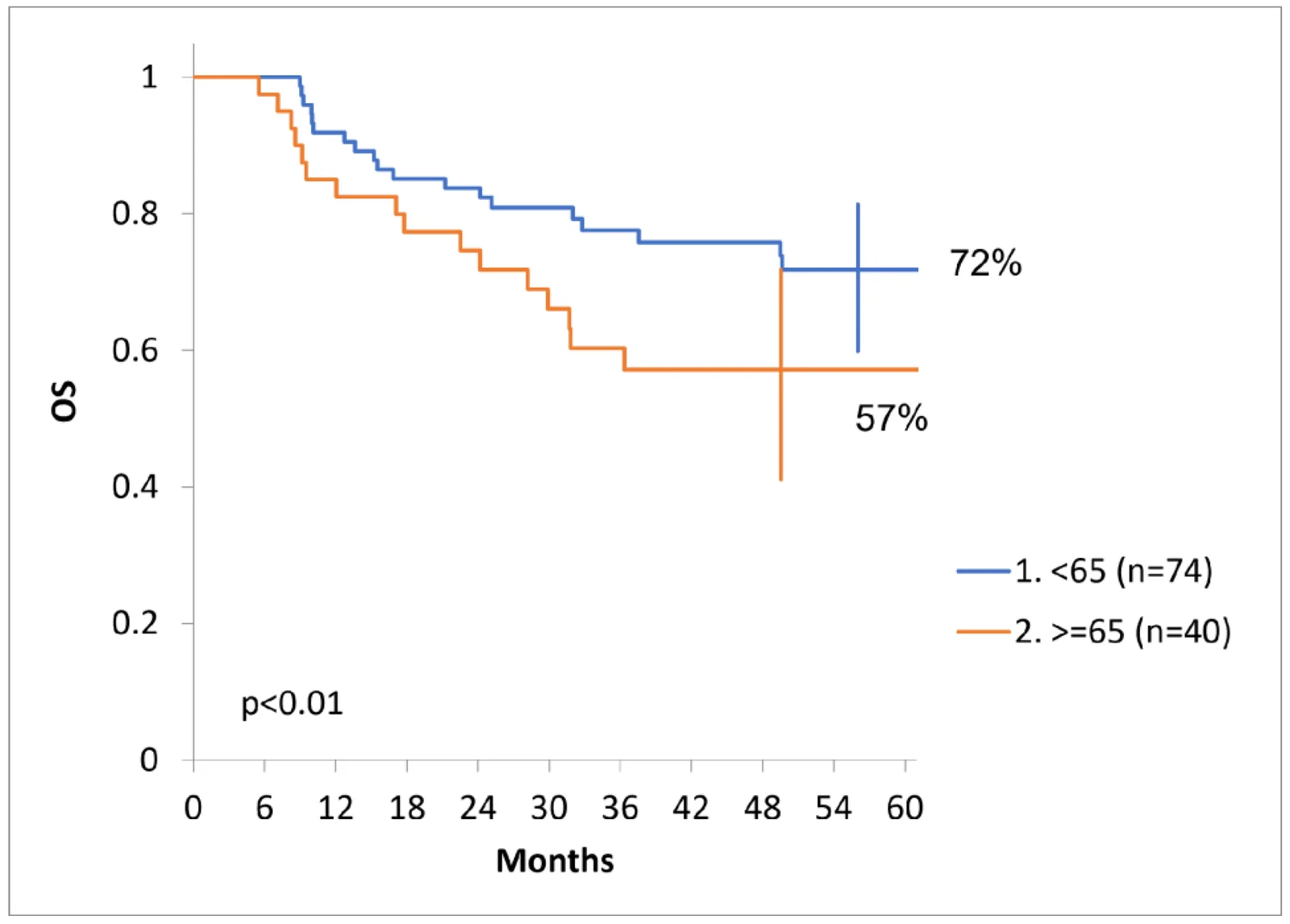
OS at 5 years according to age at diagnosis.

**Figure 3:**
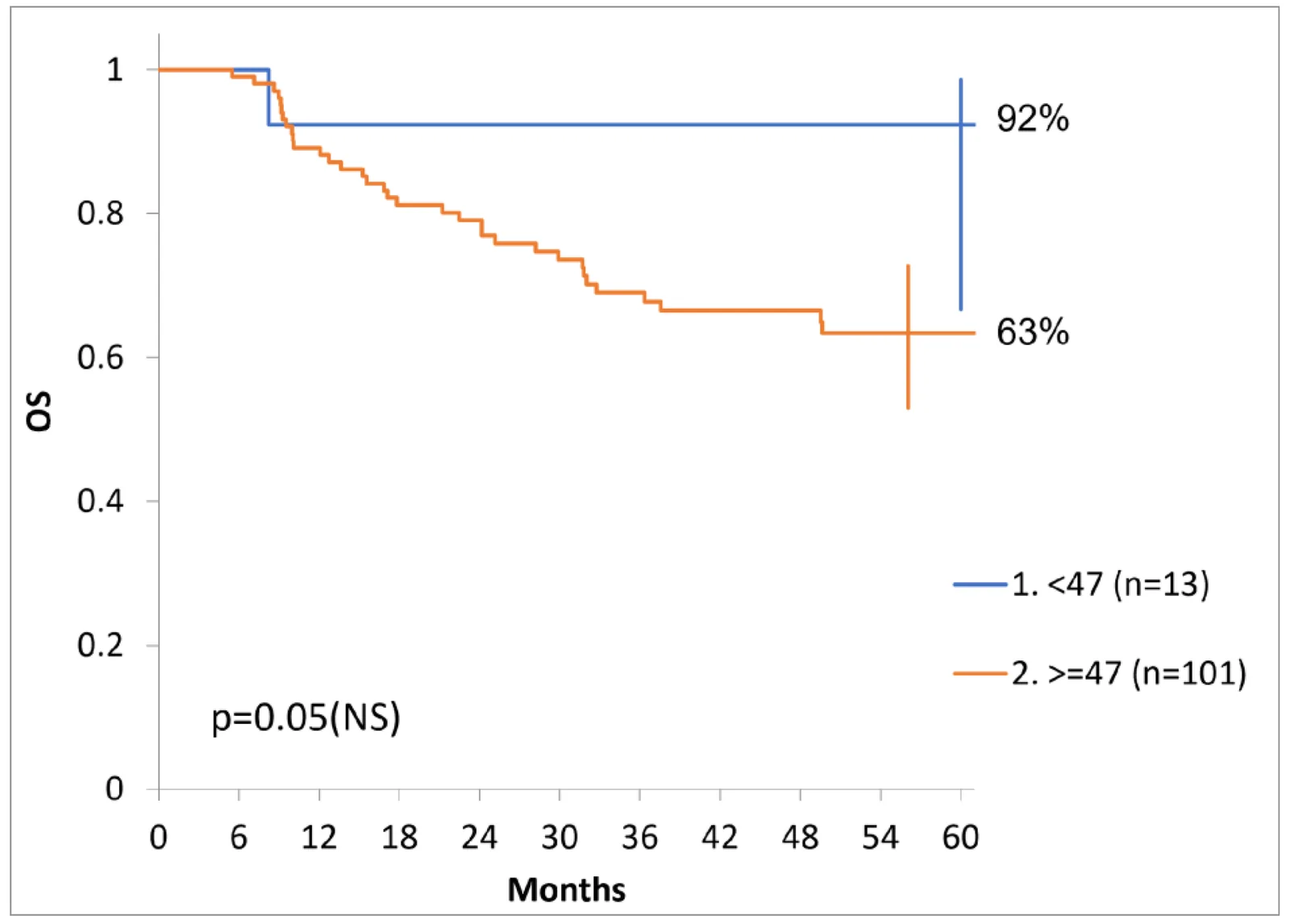
OS at 5 years according to CRT treatment time in days.

From univariate analysis, we selected the Charlson index and all independent variables having *p* ≤ 0.05, e.g., age at diagnosis, body mass index before CRT, body mass index at end of CRT, and protraction of RT in the Cox proportional hazards model. Stage at diagnosis and HPV status were not included because of the significant proportion of unknown HPV statuses, which also translate into staging bias. The ΔBMI variables were considered dependent to the BMI ones and did not qualify. By consecutive exclusion of the highest *p* value, only age at diagnosis and duration of CRT were validated by the Cox model (Figure 2 and Figure 3, Table 5).

## 4. Discussion

We analysed clinical variables and weight variation during CRT in patients with OPSCC, and we found that most patients experienced weight loss during treatment. The median loss was 5 kg, and almost a quarter lost at least 10% of their weight.

A pre-CRT BMI ≥25 and a post-CRT BMI of ≥30 had a better OS compared to other patients. A threshold of 5.5 kg loss during CRT or ≥10% from pre-CRT weight, a BMI of less than 24 (both pre-and post-CRT), or a decrease in BMI with 6% were statistically significant for predicting a worse OS at 5 years in univariate analysis. Only the age less than 65 at diagnosis and CRT duration of less than 47 days remained significant in the multivariate analysis for a good prognosis.

Weight loss during CRT is clinically common and indicates substantial treatment burden. Vangelov et al. analysed weight loss in 134 OPSCC patients who received radiation, with or without chemotherapy, definitive or postoperative. The incidence of ≥5% weight loss was 67% (higher than in our cohort, where it was 56.2%) and ≥10% weight loss in 26% (closer, but still higher than our findings of 22.8%) [16]. Another smaller cohort of HNC patients found that 60% lost > 5% during radiation therapy and that the mean loss was 5.8%, like our study [17].

Despite its frequent occurrence, the independent prognostic value of treatment-related weight loss for survival is inconsistent. In the SAKK randomized-trial cohort, pretreatment but not on-treatment loss independently predicted worse survival [18]. The recent systematic review of Wascinski et al. included eight studies involving HNC patients treated with definitive or postoperative radiation therapy. Pretreatment weight loss of more than 10% predicted lower OS, and post-treatment weight loss ≥10% was associated with worse DFS. However, most studies included in the systematic review did not find an association between weight loss during treatment and OS. In our study, a weight loss of ≥10% reached statistical significance for predicting lower OS in univariate analysis but not in the multivariate analysis [9].

In an oropharyngeal cohort nested in ARTSCAN, baseline BMI predicted 5-year overall survival; underweight and normal-weight patients had materially higher mortality than those with BMI > 25 kg/m^2^ [19]. In a separate 300-patient HPV-positive OPSCC cohort, BMI > 25 kg/m^2^ remained independently associated with longer overall survival after adjustment [20]. Our findings are in line with these studies, despite some differences in the cohort characteristics. In the ARTSCAN study, the population was slightly younger (median 58 years vs. 61 in our study), almost half of the patients received surgery (we excluded them), and half were treated with accelerated fractionation (vs. 96.5% conventional fractionation in our cohort). In the study of HPV-positive cases, 82% had a BMI over 25, whereas in our study, only about half of the patients were overweight. A ΔBMI > 1 kg/m^2^, was identified in the above-mentioned meta-analysis to predict a lower OS (9). In our study, a ΔBMI of ≥−1.5 was close to a statistically significant value, whereas a 6% decrease in BMI was significant only in univariate analysis. HPV status was not recorded at the time of diagnosis for most of our patients (N = 65; 57%) due to lack of health insurance coverage until recently. The HPV-positive cases were mostly stage II (N = 15/20), and in the HPV-negative/unknown subgroup, the predominant stage was IVA (N = 57; 60.6%). We acknowledge that we cannot perform a reliable survival correlation with HPV status while more than 50% had an unknown HPV status. With these caveats, the HPV status did not qualify for the Cox multivariate analysis model, although we do not exclude its potential prognostic value. However, this was unlikely, as the bootstrap analysis on our cohort showed a very low (3%) probability of HPV impact on 5-year overall survival.

All weight-loss-related parameters which we identified in the univariate analysis lost their significance in the multivariate analysis, probably due to the limited size of our cohort. A similar study, but including other categories of HNC patients, found that a weight loss of > 5% was significantly associated with worse disease-specific survival but not OS [21].

In our study, age ≥65 remained a significant prognostic for OS in multivariate analysis. Similarly, in a pooled analysis of 2688 patients from three phase-III NRG/RTOG studies, age ≥70 was associated with lower OS and cancer-specific survival; the OS disadvantage remained after covariate adjustment, including analyses accounting for smoking and p16 status, and was more pronounced in the combined RT–systemic therapy trials. Older adults were only 11.5% of the trial population, so the estimate still comes from selected trial-eligible patients [22].

A prolonged CRT duration was previously demonstrated as detrimental for HNC patients [23]. However, the 47-day threshold that resulted from our analysis represents the interval in which the 35 fractions can be delivered as 5 fr/week. Patients who received the treatment in a shorter interval represent a minority. Some of them had a 70 Gy/33 fr simultaneous boost regimen, and some received 6 fr/week in one or two weeks due to logistical circumstances. As a result, the finding should be interpreted with caution, as hypothesis-generating, and not supporting an accelerated schedule. A 6-fractions-per-week schedule may improve locoregional control, but a clear OS benefit has not been demonstrated in the predominantly CRT setting. When applied for RT alone, the acute morbidity increased for the 6 fr/week treatment, which can be expected to be even higher if the CRT is used [24,25].

Conventional obesity-associated insulin/IGF-1 and inflammatory pathways would more often be expected to favour tumour growth, not improve survival. However, a correlation of higher BMI with an improved survival in head and neck cancer patients was documented multiple times. The “obesity paradox” can have several explanations. Firstly, the higher-BMI patients may be better able to tolerate the acute catabolic period of mucositis, dysphagia, dysgeusia, xerostomia, and weight loss without crossing into severe malnutrition or functional decline. However, the better tolerance cannot be expected to automatically translate into a long-term survival difference. Secondly, a low or normal pre-treatment BMI may be the visible consequence of subclinical cancer burden, pretreatment dysphagia, low intake, sarcopenia, frailty, comorbidity, or systemic inflammation—not the cause of an inferior outcome. Thirdly, significant confounding factors can influence the analysis, including smoking, alcohol exposure, socioeconomic level, comorbidity, performance status, and access to care, all of which can all correlate with BMI and prognosis.

Metabolic factors that would influence tumour control, yet are unidentified, represent a topic of debate and further investigation. In a 445-patient curative definitive-CRT cohort, overweight BMI was associated not only with overall/progression-free survival but also with complete response and less locoregional failure [26]. However, the same authors analysed data from a cohort of 738 patients and concluded that being overweight was associated with a likelihood of complete response and OS but had no association with cancer-specific survival [27].

All the patients included in our study were recommended nutritional counselling. However, not all of them had the same compliance to the advised nutritional interventions, which could have had an influence on their weight variation. Food supplements are not reimbursed by the national public insurance coverage in our country, so socio-economic factors are likely to influence their accessibility. Obviously, the incidence and severity of acute toxicities influenced the food intake.

The present study has several limitations. Its retrospective, single-centre design limits generalizability. The sample size and unavailability of HPV status for all our patients restrict subgroup analyses and reduces statistical power. Alcohol consumption, details on tobacco use, performance status, and other factors which could influence survival were not evaluated. Weight could have been influenced by imprecise measurements, intensity of acute side effects, the compliance with the nutritional counselling, use of food supplements, or the presence of enteral nutrition devices. The identified significant threshold indices of body weight variation univariate analysis might be purely by chance. In addition, changes in BMI values could not distinguish between adiposity loss and sarcopenia. A retrospective study on 190 patients with HNC in which BMI and sarcopenia were assessed concluded that weight loss without skeletal muscle depletion did not affect outcomes [28].

However, we tried to minimize bias by including a group of consecutive patients from a regional reference centre, with a 5-year follow-up. They received homogenous standard-of care treatment and nutritional counselling. We extracted clinical, treatment-related, and weight variation data on which we performed univariate and multivariate analyses to further explore these associations.

The study’s novelty is represented by integration of clinical variables with dynamic weight changes. Additionally, we evaluated the role of weight loss in absolute and relative values, as well as pre- and post-CRT BMI. Moreover, to the best of or knowledge, this is the first study on this topic which reports data from Romanian OPSCC patients who received CRT with curative intent. We will continue to look at the BMI variation post- versus pre-treatment as a potential prognostic factor in multivariate analysis on a larger cohort of patients.

## 5. Conclusions

Patients with OPSCC treated with concomitant CRT experience significant weight loss during treatment. BMI and weight loss were associated with 5-year overall survival in univariate but not in multivariate analysis on our homogenously treated 114-patient cohort. Younger age and shorter treatment time were significant predictors for better survival. Prospective analysis incorporating baseline and longitudinal lean body mass, fat distribution, inflammation, pretreatment weight loss/dysphagia, feeding-tube use, treatment interruptions, HPV status, smoking, and nutritional information could enable a more refined analysis and potential nutritional variation biomarkers.

## Figures and Tables

**Table 1:** Patients’ baseline characteristics.

Variable	Value	%
**Gender (N = 114)**		
Female	22	19.3
Male	92	80.7
**Age (N = 114)**		
Median (range)	61 (38–78)	
**Charlson Comorbidity Index (N = 114)**		
<5	81	71.1
≥5	33	28.9
**Current smoker (N = 114)**		
Yes	64	56.1
No	50	43.9
**Subsite (N = 114)**		
Tonsil	69	60.5
Base of tongue	20	17.5
Posterior pharyngeal wall	3	2.6
Soft palate	14	12.3
Vallecula	5	4.4
**Grade (N = 114)**		
G1	12	10.5
G2	61	53.5
G3	21	18.4
NA	20	17.5
**HPV status (N = 114)**		
Negative	29	25.4
Positive	20	17.5
NA	65	57.0
**Staging**		
**HPV+ cases (N = 20)**		
Stage II	15	75
Stage III	5	25
**HPV−/Unknown cases (N = 94)**		
Stage II	2	2.2
Stage III	29	30.9
Stage IVA	57	60.6
Stage IVB	6	6.4

NA—not available.

**Table 2:** Treatment characteristics.

**RT Dose (N = 114)**		
70 Gy/35 fr (7 weeks)	97	86.6
70 Gy/33 fr (6.5 weeks)	12	10.5
Other (65–76 Gy/30–38 fr)	5	5.2
**Type of concomitant ChT (N = 114)**		
Cisplatin 100 mg/mp every 3 weeks	65	57.0
Cisplatin 40 mg/mp weekly	26	22.8
Cetuximab 250 mg/m^2^ weekly (after loading dose 1 week prior RT)	7	6.1
Carboplatin AUC2 weekly	5	4.4
Other/combinations	11	9.6

Legend: RT—radiation therapy, ChT—chemotherapy, G—grade, AUC—area under curve, NA—not available.

**Table 3:** Estimated 5-year OS according to clinical variables.

		n	5 yrs OS (%)	OS CI (%)	HR	HR CI	*p*
**Gender**	F	22	69.3%	43.3–87.0%	1.63	1.21–2.20	0.21
	M	92	65.5%	54.8–74.8%			
**Age (years)**	<65	74	71.8%	56.0–81.4%	2.47	1.96–3.11	**0.01**
	≥65	40	57.2%	41.3–71.8%			
**Stage**	II	17	88.2%	65.7–94.1%	2.72 (II vs. III + IV)	1.59–4.66	0.12
	III	34	66.2%	48.2–79.8%	2.34 (II + III vs. IV)	1.53–3.58	0.31
	IV	63	61.6%	48.3–73.3%			
**HPV**	NA	65	69.4%	56.9–79.5%	1.32 *	0.98–1.79	0.69
	−	29	55.0%	35.3–73.3%	0.84 ** 0.69 ***	0.55–1.28 0.41–1.15	0.46 0.43
	+	20	72.7%	49.2–88.0%			
**Charlson index**	<5	81	66.6%	54.8–76.7%	1.3	1.0–1.6	0.45
	≥5	33	65.3%	47.8–79.5%			
**Current smoker**	Yes	64	66.6%	53.4–77.1%	0.80	0.7–1.0	0.55
	No	50	68.0%	52.2–79.74%			
**RT overall time (days)**	<47	14	92.3%	66.7–98.6%	2.52	1.62–3.90	**0.05**
	≥47	100	63.4%	53.0–72.7%			

Legend: OS—overall survival, HR—Hazard ratio, CI—confidence interval, HPV—Human Papilloma virus, CRT—concurrent chemoradiation, * = comparing NA vs. HPV(+) combined with HPV(−), ** = comparing NA and HPV(−) vs. HPV(+), *** HPV(+) vs. HPV(−). Significant *p*-values are bolded.

**Table 4:** Estimated 5-year OS according to pre-CRT and post-CRT BMI, BMI difference, and percentage of weight loss during CRT.

		n	5 yrs OS (%)	OS CI (%)	HR	HR CI	p
**Weight loss#**	<5%	51	75.7%	57.1–82.9%	1.56	1.29–1.88	0.15
	≥5%	63	62.4%	49.3–73.9%			
**Weight loss#**	**<10%**	**88**	**71.6%**	**60.8–80.4%**	**2.28**	**1.70–3.05**	**0.03**
	**≥10%**	**26**	**50.0%**	**30.8–69.1%**			
**Weight loss#**	**<5.5 kg**	**72**	**75.7%**	**63.8–84.6%**	**2.19**	**1.77–2.71**	**0.02**
	**≥5.5 kg**	**42**	**51%**	**35.4–66.4%**			
**pre-CRT BMI**	<25	47	61.9%	47.0–74.8%	0.70 *	0.56–0.88	0.53
	25–29.9	49	71.2%	56.6–82.4%	0.70 **	0.47–1.05	0.64
	≥30	18	66.7%	39.6–86.0%			
**pre-CRT BMI**	**<24**	**38**	**55.6%**	**39.3–70.8%**	**0.50**	**0.40–0.63**	**0.04**
	**≥24**	**76**	**72.6%**	**60.8–81.9%**			
**post-CRT BMI**	<25	72	61.1%	49.2–71.8%	0.68 *	0.53–0.87	0.25
	25–29.9	30	74.0%	51.9–88.3%	0.50 **	0.31–0.81	0.12
	≥30	12	83.3%	55.2–95.3%			
**post-CRT BMI**	**<24**	**59**	**56.1%**	**43.1–68.4%**	**0.40**	**0.33–0.48**	**0.01**
	**≥24**	**55**	**78.7%**	**64.5–88.3%**			
**Δ BMI**	<−1.5	55	59.8%	44.0–71.1%	0.56	0.46–0.67	0.06
	≥−1.5	59	74.3%	61.1–84.2%			
**Δ BMI** (%)	**≤6%**	**60**	**74.6%**	**61.5–84.4%**	**0.51**	**0.41–0.61**	**0.03**
	**>6%**	**54**	**57.6%**	**43.3–70.6%**			

Legend: OS—overall survival, HR—hazard ratio, CI—confidence interval, BMI—body mass index, CRT—chemoradiotherapy, Δ BMI—BMI decrease during CRT, # Weight loss during CRT; * <25 vs. ≥25; ** <30 vs. ≥30.

**Table 5:** Cox proportional hazards model analysis.

**Covariate (START)**	**b**	**SE**	***p***	**HR**	**95% CI of HR**
**AGE at DIAGNOSTIC**	0.05169	0.021649	0.05103	1.053	1.000–1.108
**Charslon Index**	−0.02269	0.1497	0.8795	0.978	0.7300–1.309
**IMC-PRE**	0.1598	0.0953	0.9382	1.173	0.9742 = 1.4130
**IMC_POST**	−0.2231	0.1016	0.02816	0.8	0.6562–0.9754
**RT overall treatment time**	0.1039	0.03695	0.00494	1.11	1.0323–1.1923
**Covariate (END)**	**b**	**SE**	***p***	**HR**	**95% CI of HR**
**AGE at DIAGNOSTIC**	0.04776	0.02135	0.02533	1.049	1.0061 to 1.0935
**RT overall treatment time**	0.1039	0.03569	0.00359	1.11	1.0349 to 1.1895

Legend: b—regression coefficient, SE—standard error, *p*—statistical significance.

## Data Availability

The original contributions presented in this study are included in the article. Further inquiries can be directed to the corresponding authors.
